# Making Medical Education Socially Accountable in Australia and Southeast Asia: A Systematic Review

**DOI:** 10.1007/s40670-025-02322-x

**Published:** 2025-02-25

**Authors:** Jyotsna Rimal, Ashish Shrestha, Elizabeth Cardell, Stephen Billett, Alfred King-yin Lam

**Affiliations:** 1 School of Medicine and Dentistry, Griffith University, Gold Coast Campus, Southport, QLD 4222 Australia; 2 Koirala Institute of Health Sciences, B.P, Dharan, Nepal; 3 School of Health and Medical Sciences, University of Southern Queensland, Ipswich Campus, Ipswich, QLD 4305 Australia; 4 School of Education and Professional Studies, Griffith University, Mount Gravatt Campus, Mount Gravatt, QLD 4122 Australia

**Keywords:** Social accountability, Contextual learning, Community-based education, Graduate outcomes, Partnership, Universal health coverage, Sustainable development goals

## Abstract

Social accountability (SA) measures are critical in medical education. A systematic review was conducted following PRISMA-systematic review guidelines, across Australia and Southeast Asia Region (SEAR) countries to identify their purposes, practices, experiences, and recommendations. Four electronic databases were searched using search terms and key themes were identified through inductive analysis. Fifteen included studies identified five themes: (i) social obligation spectrum, (ii) learning environment, (iii) values of SA, (iv) graduate outcomes, and (v) partnerships. The cross-cutting themes were governance, education, service, and research. This review highlighted the role of SA in medical education and identified themes applicable for Australia and SEAR countries.

**Supplementary Information:**

The online version contains supplementary material available at 10.1007/s40670-025-02322-x.

## Introduction

Social accountability (SA) of medical schools is defined as an obligation to direct their education, research, and service activities towards addressing the priority health concerns of the community, region, and/or nation they have the mandate to serve [1]. Globally, identifying and enacting SA measures in medical education is becoming a critical component of their regulation and accreditation to address societal and health system inequities [2–4]. A medical school may be across the spectrum of the social obligation scale, from being responsible, to responsive, to being socially accountable [5]. Social accountability as an indicator of excellence is critical to achieving sustainable development goals (SDGs) by addressing health inequities for producing socially responsible professionals committed to improving public health outcomes as highlighted by the 2030 Agenda for SGDs and universal health coverage (UHC) [6–9]. This lays a strong foundation to include SA values in health professions educational institutions (HPEIs) in curriculum and governance, stakeholders’ engagement, societal impact, environmental accountability, implementation research, etc. [10, 11]. Frameworks such as the Institutional Self-assessment SA Tool (ISAT); Conceptualization Production Usability (CPU) model; Clinical activity, Advocacy, Research, Education, and training (CARE) model; and their strategic directions encouraging compliance with SA principles are providing the basis for these components [3, 12, 13]. Socially accountable medical education (SAME) may address community health needs by adopting community-based education through a Pentagram partnership [1]. With all the imperatives, the health outcomes of the community, based on investment in these educational activities, are still limited [14, 15]. Addressing social accountability in medical education is a complex endeavour, and several knowledge gaps persist in this area. Concept and scope are ambiguous of social accountability which hampers the development of standardised frameworks for the implementation [16]. Integrating SA into medical curricula in a sustainable and contextually relevant manner remains a challenge [17].

The underdevelopment of structured frameworks for involving communities as active partners in medical education is an area for further exploration [18]. Understanding how locally trained, socially accountable physicians can address global health challenges is also limited. Another important knowledge gap area is how educational efforts of medical schools are linked to tangible health system improvements [17, 18]. In Australia, SA is mandated by the Australian Medical Council (AMC) through its SA committee [19]. Evidence for SA mandate in World Health Organization South-East Asia Region (WHO-SEAR) countries is unknown. This led our team to work on a research question ‘What makes the medical education in Australia and WHO-SEAR countries socially accountable?’ This systematic review aimed to identify themes and document evidence on SA domains applied in medical education in these countries.

## Materials and Methods

The review was conducted to identify gaps and potential SA measures in medical education and to categorise the themes for medical programmes. We used the five-stage framework of Arksey and O’Malley (2005) to map the key concepts underpinning the research question [20]. The PRISMA statement 2020 for systematic review guided its reporting [21]. At the outset, a protocol for systematic review was developed to map and summarise the purposes, practices, experiences, and recommendations. To identify and delineate eligible articles, four key databases, EMBASE, SCOPUS, Medline Via Ovid, and ERIC, were selected. Search terms were carefully chosen (Table 1) to permit the comprehensiveness of data collection published until the date of the final search (October 30, 2022). Additional searches were further conducted in December 2023 with no new studies identified. The search terms used were (i) the social obligation scale as defined by Boelen (1995) or other related terms, (ii) medical programme or other related terms, and (iii) Australia and WHO-SEAR countries, as shown in Table 1. The search string was created using ‘OR’ and ‘AND’ to combine these search terms.

**Table 1:** Search terms and the search string used for the selected databases

OR		OR		OR
Social accountab* Social responsibility Social responsiveness Social mission Social change Social justice Societ* needs	AND	Medicine Health Professions Education Program School Curriculum	AND	Australia Bangladesh Bhutan North Korea India Indonesia Maldives Myanmar Nepal Sri Lanka Thailand Timor-Leste

*Denotes related morphemes

To capture purposes, approaches, and practices, eligible articles included original peer-reviewed journal publications in English, from Australia or one of the WHO-SEAR countries. Articles were excluded if they did not fulfill study objectives or were conference proceedings or abstracts due to their limitation for thematic analysis. Systematic or narrative reviews were also excluded; however, the reference list of relevant original research was hand-searched for any additional article meeting the inclusion criteria. Identified records were uploaded into COVIDENCE, duplicates were removed, and data was extracted.

### Quality Assessment and Data Extraction

After the selection of articles, two authors (JR and AS) independently extracted data into the COVIDENCE template which was contextualised to address the research question. The contextualisation assisted in deriving the understanding of the purpose, practice, experience, and recommendation of SA. The data collection process involved independently completing the data extraction sheet by two authors (JR and AS) for titles and abstracts to determine the eligibility of studies to build up inter-rater reliability. If eligibility could not be determined with the title and abstract then, the full article was referred to. Discrepancies in selection were resolved through moderation amongst three reviewers before data extraction. The quality assessment template was adopted as per the critical appraisal criteria as shown in supplementary document 2, The criteria included (i) clarity and relevance of research questions, (ii) study design and its appropriateness for addressing the research question, (iii) selection of study participants and its appropriateness, (iv) presentation of the impact of sample population, (v) appropriateness of study questions, (vi) inclusion of studies strength and limitation, and (vii) recommendation for future study [22]. Chart discrepancies were resolved by consensus. After the data collection process, data charting and synthesis were initiated.

### Data Charting and Synthesis

Data charting was used to identify, characterise, and summarise evidence on SA and was managed in an electronic Excel spreadsheet. Multiple iterative rounds of coding were completed to capture a variety of perspectives, to expand the range of developed concepts, and to understand their properties and relationships. An inductive analysis approach was applied which built-up codes from texts to produce an understanding of content. The authors (JR, AS, EC) engaged with the texts to identify the basis for developing the initial coding schema. In this first round of coding, the authors charted the research question-related data, i.e. purpose, practice, experience, and recommendations. One author (JR) grouped the charted codes into initial categories and agreed upon by other authors (AS, EC). At the outset of the second round of analysis, the authors formulated a description/definition of the themes based on relationships and links between codes. The final themes were derived from these codes. Following the second round of analysis, the results were synthesised and interpreted.

## Results

**Fig. 1:**
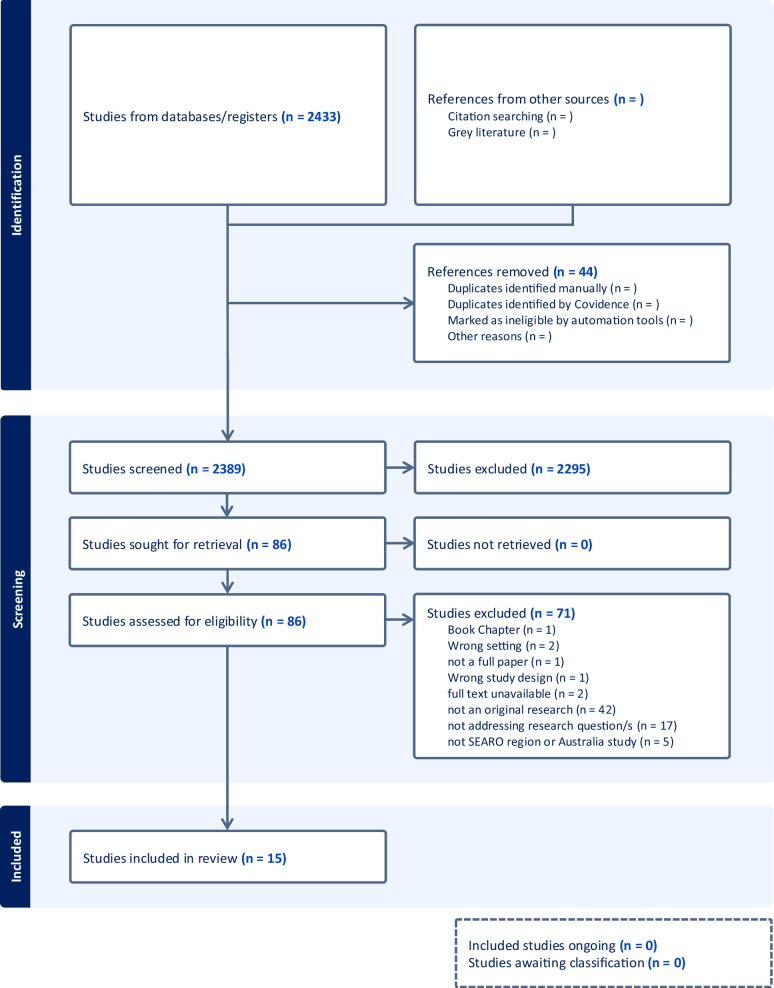
PRISMA chart of database search and record selection for the systematic review

A total of 2433 articles and studies were screened. Due to irrelevancy to study objectives, 2295 were removed and 86 full-text studies were assessed for eligibility based on inclusion criteria. Seventy-one studies were excluded for multiple reasons (Fig. 1), qualifying 15 studies for the final review. The PRISMA chart of database search is shown in Fig. 1. The countries represented were Australia (11), India (2), Nepal (1), and Thailand (1). The articles comprised 4 quantitative, 9 qualitative, and 2 mixed-method studies. The summary of all included studies and quality assessment can be availed from supplementary documents 1 and 2, respectively. Missing result on quality assessment is also reflected in supplementary document 2.

Seven descriptive themes emerged from the codes generated by the first level of analysis. These descriptive themes were condensed into five domains, namely (1) social obligation spectrum (SOS), (2) learning environment, (3) values of SA, (4) graduate outcomes, and (5) partnership (Table 2). SAME emphasised aligning medical education, research, and service with societal needs, focussing on social values, justice, and community responsibility. It integrated inter-professional education, cultural competencies, community engagement, and personalised learning environments to prepare socially accountable graduates. SAME prioritised equitable healthcare by addressing healthcare disparities, emphasising rural and indigenous health, and fostering community-targeted problem-based learning (PBL). Governance, accreditation, and partnerships with stakeholders like policymakers, NGOs, and health providers were drivers for the implementation of socially relevant curricula and policies. Graduates trained in SAME demonstrated enhanced societal alignment, equity, and cost-effective healthcare delivery, contributing to workforce retention and leadership in underserved areas. Key cross-cutting themes—education, governance, service, and research—are interconnected to produce transformative health professionals committed to addressing priority health and societal needs. The details of the results are discussed under five domains of the themes that emerged from analyses of SAME in Australia and SEAR countries.

**Table 2:** Codes, categories, and themes after two levels of analysis

SN	Codes and categories	Themes
1	Social mission, social justice, social need, social values, giving back	SOS
2	Inter-professional education, professionalism, cultural competencies, teachers as change agents, changing roles of doctors/medical schools, health advocacy, stakeholders (community, students, policymakers) engagement, and community-targeted problem-based learning	Learning environment
3	University vision, mission statements (research, service, education), curriculum, quality service delivery, accreditation system	Values of SA (quality of education and care)
	Care to understand, priority healthcare research, health equity, community engagement, communities’ or societal health needs and voices, student selection	Values of SA (equity)
	Socioeconomic status, work settings, cultural competencies, community placements, community participation, quality service delivery	Values of SA (relevance)
4	Workforce needs, work-ready graduates, graduate practice retention, graduate competencies, graduate output/impact	Graduate outcome
5	Formal linkages with government and other institutions, professional associations, collaboration, educational administration, regulatory bodies, consortiums, societies	Partnership

### Domain 1: Social Obligation Spectrum

**Fig. 2:**
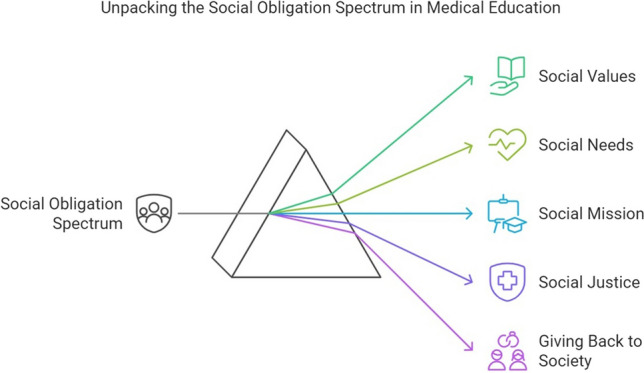
The range of the social obligation spectrum

The SOS encompassed social values, social needs, social mission, social justice, and giving back to society, with awareness amongst medical students and staff directly impacting community health outcomes (Fig. 2) [23–29]. Medical school’s social missions included providing quality staff for healthcare, more general practitioners, improving access for indigenous people, reflecting their responsibility to the community, and awareness of health needs [25, 30]. The mission influenced the programme outcomes and internalised social values in students affecting their career choices and practice approaches [24, 25]. The six key domains of the social mission were community responsibility, establishment influence, locality influence, focus on general practice, rural responsibility, and indigenous health need awareness [30]. Social justice awareness was central to students’ understanding of their roles [25, 31]. Recognising social and cultural values emphasised SA in health outcomes, underscoring the importance of giving back to society [24, 32, 33]. Therefore, medical schools should align their education, research, and service to address aspects of SOS, which are reflected in their learning environment.

### Domain 2: Learning Environment

The SAME emphasised personalised interactions amongst educators within a socially constructed learning environment focussing on key codes such as inter-professional education (IPE), professionalism, cultural competencies, and teachers as change agents. Inter-professional learning environments, where students learn in teams from diverse professional backgrounds, were crucial for achieving educational outcomes [28, 34]. Sustaining community activities as part of the learning process was essential for developing SA competencies [27]. Cultural competencies, encompassing values, backgrounds, differences, contexts, and cultural complexities, were vital, with educators’ cultural sensitivity and awareness laying a strong foundation for the learning environment [24, 25, 28, 30, 35]. ‘Teachers as change agents’ and ‘enlightened change agents’ were crucial as role models to prepare graduates with leadership skills within the health system [24, 35]. Effective SA relied on integrating new means of interaction, such as street plays, community partnerships, health camps, and student engagement [24]. Student engagement in community action projects fostered authentic learning, enhancing their understanding and commitment to the mission, particularly through practical experience with underserved populations [25, 35]. Thus, curricula in SAME of the included studies were community-oriented, interdisciplinary, and integrated, often incorporating community-targeted PBL to prepare students for real-world health challenges [23–25, 27–35]. This approach aligned with the values of SA by preparing students to address community health needs effectively [23, 34].

### Domain 3: Values of SA

Explicit reference to the values of SA was a basis for the conceptualisation of its accountability [1]. The codes and their components are depicted in Tables 2 and 3 respectively. The SAME aligned their vision and mission statements towards society based on their school’s philosophy [24, 25, 29, 30, 32, 35]. Components of quality of education, quality health services, equity, and relevance are shown in Table 3 and are described below under quality of education and healthcare services, equity and relevance, and cost-effectiveness for the purpose of clarity.

**Table 3:** Components identified for quality education, quality health services, equity, and relevance

Quality of education
Contextualised community-based curriculum in alignment with its mission, vision, and goals
Alignment of contextual influence of values and mission with SA
Students’ immersion in an authentic clinical environment in a community setting through workplace-based training
The accreditation system is crucial to motivating medical schools towards SA
World Federation for Medical Education accreditation aligns towards SA
Quality health services
Institutional commitment to health services
Engaging and supporting community health service providers as educators
Innovative solution to priority health and health service problems
Need-based research; action-oriented participatory research, cross-disciplinary multi-institutional collaborative research priority agenda
Student engagement in healthcare services through service learning
Equity
Students’ involvement in rural healthcare
The gap between intended and enacted curriculum needs to be bridged
Connecting personal and population health
Student recruitment from rural and underserved communities
Educators walking the talk
Involvement of community leaders in engaging the community
Learning in context through community placements
Relevance
Support for students with low socio-economic status
Meaningful dialogues amongst stakeholders for socio-cultural appropriateness
Graduates connecting personal and population health
Students work with the community in planning, implementing, and evaluating programmes
Awareness of social and cultural values helps students to give back to the community
Health promotion activities for culturally appropriate, affordable, and innovative solutions
Community placement: an opportunity to realise the relevance of learning
Continuity of community and clinical experience throughout the programme
Partnership with stakeholders

#### Quality of Education and Healthcare Services

Quality education and healthcare in SAME relied on their visions, missions, curricula, and accreditation standards. Governance shaped community-oriented curricula, integrating PBL, rural and indigenous health training, and international electives [30]. Accreditation standard was a driver for societal alignment, fostering care for underserved populations and sustainable health improvements [25, 35]. Collaborative faculty, research, and student-community engagement enhanced societal transformation and policy reform. Accreditation emphasised societal obligations as measures of excellence, bridging educational impact and societal alignment [25, 30, 35].

#### Equity

The SAME prioritised addressing healthcare inequities by aligning education, research, and service with underserved populations’ needs [33]. Strategies included community engagement, contextual curricula, participatory research, and recruiting students from underserved areas [23, 24, 32]. Challenges like funding constraints, urban-centric biases, and hidden curricula persisted [35]. SAME emphasised culturally appropriate solutions, policy impact, and fostering rural healthcare understanding, aiming to prepare graduates for equitable healthcare delivery and societal needs [28, 33, 35].

#### Relevance and Cost-Effectiveness

The SAME emphasised relevance and cost-effectiveness by addressing socio-economic and cultural determinants, prioritising students from low-income backgrounds, and fostering equitable health improvements [27]. Strategies included PBL, IPE, rural placements, and culturally appropriate training [23, 29]. Stakeholder collaboration ensured community engagement and practical, socially accountable experiences [29, 35]. Governance-supported policies are required for meaningful partnership and to inspire graduates for addressing diverse health needs through affordable and innovative approaches [27, 29, 35].

### Domain 4: Graduate Outcomes

The SAME addressed workforce, community, and health needs through their nature and content, reflecting personal responsibility and values [29, 33]. Positive workforce outcomes rely on proper student selection, support, curriculum design, role modelling, and postgraduate pathways [29]. Social accountability in medical education led to higher retention rates than in traditional schools [28, 33, 36]. Graduate competencies for SA values were recommended alongside biomedical competencies with graduate teaching or preceptor roles as ways to give back [33, 37]. Success was measured by impacts on other institutions through curriculum replication and graduates becoming academic leaders [32]. Partnerships with health organisations enhanced these outcomes by aligning educational efforts with community health needs.

### Domain 5: Partnership

The SAME regularly assessed the priority healthcare needs of the community, region, and nation by collecting data and consulting with representative groups including politicians, physicians, consumers of healthcare, and government policymakers [23]. Partners for SAME included governmental and non-governmental organisations, regulatory bodies, healthcare policymakers, medical associations, students’ groups, local health providers and agencies, community centres, community partners, and educational administrators [23–25, 27, 28, 32–35]. Engaging with the government was the key to nurturing partnerships [24]. Meaningful collaboration with stakeholders and partners was considered an important commitment to addressing priority health and social needs [24, 27, 28, 32, 33, 35]. Health professionals working collaboratively with an interdisciplinary approach to teaching primary healthcare was encouraged [34]. Governance and curriculum decisions needed to be made collaboratively, involving stakeholders and partners [28]. Therefore, it was recommended that SAME should undertake collaborative research projects to ensure their programmes align with community health priorities [28].

### Cross-Cutting Themes

**Fig. 3:**
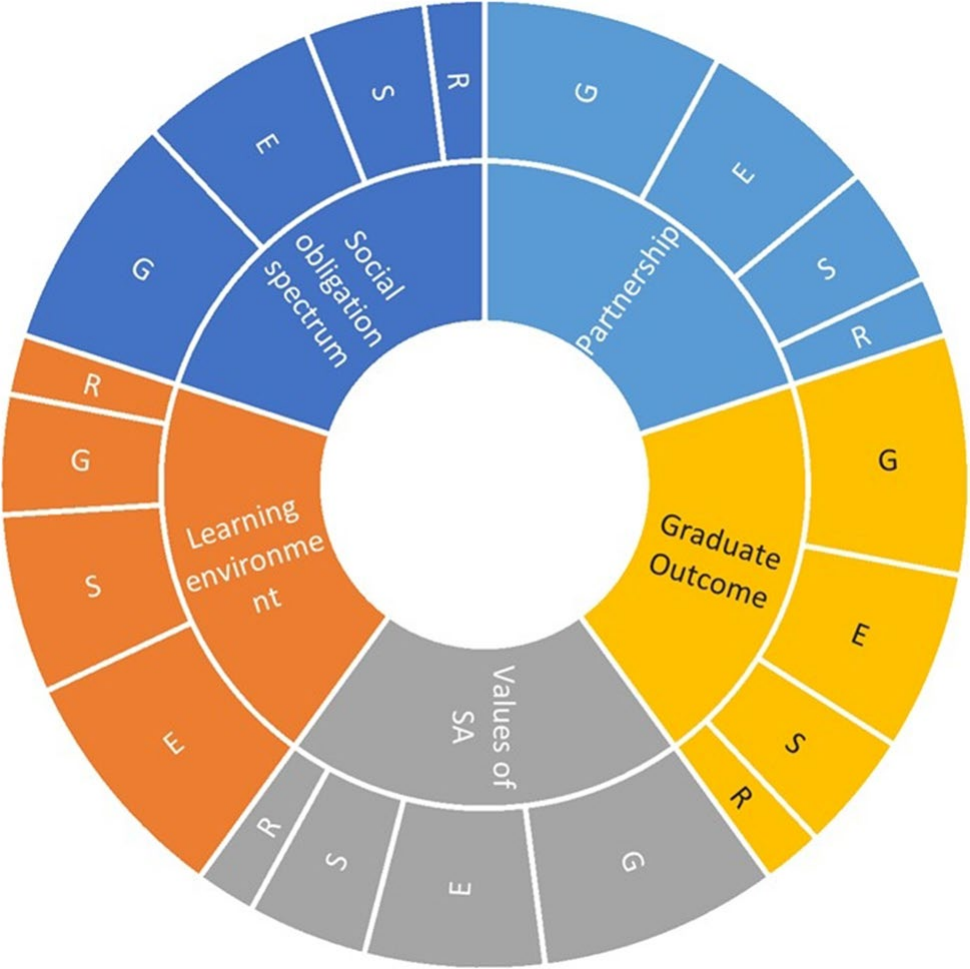
Map of SA themes by category and their interrelationship. The alphabets represent the cross-cutting (horizontal) themes as G, governance; E, education; S, service; and R, research

Categories of cross-cutting (horizontal) themes included education, governance, service, and research in producing socially responsible healthcare professionals as shown in Fig. 3. Across the four themes, governance was considered of utmost importance except for the learning environment where education covered the maximum proportion. Education was the second most important cross-cutting theme followed by service and research. Governance had the least influence on the learning environment, potentially allowing educators to create student learning environments without governance constraints. Education covered the maximum proportion of the learning environment, directly correlated with curriculum design, teaching methods, and the roles of educators. This theme also highlighted the impact of education on student competencies and professional development. Service was the cornerstone of SAME, where service activities contributed to community health and enhanced student learning. The final cross-cutting theme, research, was crucial for knowing what worked and what did not. Evidence-based approaches are instrumental at all levels of medical education in advancing medical knowledge and practice. Need-based, action-oriented participatory approaches may be adopted for addressing SA in medical education. All these cross-cutting themes interconnected and supported each other across all the vertical themes of SAME. A holistic approach was desired to ensure comprehensive medical education and community health impact.

## Discussion

This systematic review aimed to identify themes in SA and document evidence on policy, practice, experience, and recommendation in Australia and WHO-SEAR countries. There was limited evidence for SAME within WHO-SEAR countries, suggesting the need for future research in the area. In contrast, Australian medical schools demonstrated a commitment to SA, supported by the Australian Medical Council’s policy mandate [19]. Eleven included studies highlighted SA efforts in Australian medical schools.

All medical schools are fundamentally responsible for the communities they serve by ensuring the quality of education [3, 38]. The identified themes may influence the planning, implementation, and evaluation of medical education programmes, shaping the philosophy of medical schools [39–45]. The following four key areas are being reflected upon along with the cross-cutting themes from wider perspectives.

### Key Area 1: Social Obligation Spectrum and Community Impact

The integration of social values and missions in medical education influences community health outcomes and career choices which is essential for advancing UHC and SDGs by ensuring that medical schools produce healthcare professionals attuned to community needs, improve access to care for marginalised populations, and address social justice in health outcomes [9]. The social mission of medical schools includes providing quality staff for healthcare, more general practitioners, increasing access for indigenous people, medical schools’ responsibility to the community, awareness of indigenous health needs, improvement of rural health, and sensitising stakeholders to social obligation [25, 30, 46].

### Key Area 2: Socially Accountable Learning Environment

The socially accountable learning environment emphasises active learning approaches like PBL, IPE, professionalism [47–51], cultural competencies [52, 53], and the role of teachers as change agents and community engagement [32, 40] and is essential for developing SA competencies. These competencies are crucial for advancing UHC and achieving SDGs by preparing graduates to address the needs of underserved populations and promote equitable access to quality care [9, 54]. Effective SA also relies on innovative interactive methods such as street plays, community partnerships, health camps, and action projects providing practical experience with underserved populations [40, 55–57], enhancing students’ understanding and commitment to the social mission [58, 59].

### Key Area 3: Quality Education and Health Services

Quality education that incorporated community-based curricula [41, 55, 57], workplace-based training, and equity-focussed recruitment are key to achieving UHC and SDGs. This ensures healthcare professionals deliver equitable, relevant, and high-quality services [9, 51, 58, 60]. This involves institutional commitment, student involvement in rural healthcare, support for underserved communities, and continuous community and clinical experience [50, 51].

### Key Area 4: Graduate Outcomes and Partnerships

Tracking graduate outcomes and fostering partnerships with health organisations and community stakeholders align medical education with community health needs, contributing to UHC and SDGs by ensuring that graduates are prepared to address complex health challenges and provide equitable, high-quality care through an interdisciplinary approach [9, 40, 41, 46, 51, 56, 61, 62]. Regular assessment of community health needs and meaningful dialogue with representative groups is crucial in informing medical school curricula and activities [40].

### Cross-Cutting Themes

The cross-cutting themes highlighted the importance of governance, quality education, service, and research in producing socially responsible healthcare professionals. The interconnected themes are essential for advancing UHC and SDGs by ensuring that healthcare professionals are socially responsible, equipped with relevant competencies, and capable of improving community health through evidence-based equitable practices. Governance plays a key role in education in terms of policymaking, accreditation, and institutional accountability, influencing educational outcomes, service quality, and research integrity. Governance influences educational outcomes, service quality, and research integrity through policymaking, accreditation, and institutional accountability [23, 24, 28, 30]. Education is linked to curriculum design, teaching methods, and assessment [27]. Service activities and research enhance community health and student learning [50, 51] and provide evidence-based research priorities [35].

### Limitation

The studies presented several limitations that need to be addressed in future research. Biggs (2011) suggested further investigation to evaluate the impact of government strategies aimed at increasing indigenous doctors and rural practitioners [30]. Chapagain (2000) noted the potential biases in faculty perceptions and emphasised the need for larger sample sizes to validate findings [23]. Dandekar (2021) highlighted participant self-consciousness in interviews and limited generalisability due to the small sample size [24]. Ellaway (2018) acknowledged the contextual nature of their study, calling for further research to measure the student experience and mission translation [25]. Larkins (2018, 2013) emphasised difficulties in data collection across diverse contexts and schools, suggesting the need for capacity-building and longitudinal tools [26, 35]. Ross (2014) pointed out internal bias, especially in schools where evaluators were also involved in framework design, recommending peer review processes [28]. Woolley (2019, 2018) discussed potential biases in supervisor evaluations of graduates and limitations in survey response rates, impacting its generalisability [36, 37]. These limitations collectively highlighted the need for larger, more diverse samples, external peer reviews, and longitudinal data collection to enhance the validity and applicability of findings.

A limitation in our review was the inability to segregate country-specific SA measures, particularly in SEAR countries. This challenge was due to the limited availability of studies on individual countries within the region. Future research should aim to address this gap by conducting country-specific studies to enable a deeper understanding of SA efforts and challenges within each country and their context.

## Conclusion

This systematic review has identified key factors related to social accountability and has underscored its critical role in medical education across Australia and WHO-SEAR countries. This work has highlighted the critical role of vertical themes of the social obligation spectrum, learning environment, values of SA, graduate outcomes, and partnership linked with the horizontal themes of governance, education, service, and research. Their interconnectedness enhances community health outcomes, aligns medical training with societal needs, and fosters the development of competent, socially responsible healthcare professionals, in Australian and WHO-SEAR contexts.

## Supplementary Information

Below is the link to the electronic supplementary material.

Supplementary file1 (DOCX 32.1 KB)

Supplementary file2 (DOCX 18 KB)

## Data Availability

The authors confirm that the data supporting the findings of this study are available within the article [and/or] its supplementary materials.
