# Supplementary material for: Making Medical Education Socially Accountable in Australia and Southeast Asia: A Systematic Review
Source: Med Sci Educ. 2025 Feb 25;35(3):1767–76. doi: 10.1007/s40670-025-02322-x (PMC12228936; doi:10.1007/s40670-025-02322-x)
Supplement: Supplementary file 1 — Supplementary file1 (DOCX 32.1 KB) [file 40670_2025_2322_MOESM1_ESM.docx]

| **SN** | **Title** | **Country of origin** | **University** | **Year** | **Aim of study** | **Study conclusion** |
| --- | --- | --- | --- | --- | --- | --- |
| 1 | The social mission of Australian medical schools in a time of expansion. (Biggs & Wells, 2011) | Australia | Australian National University | 2011 | The expansion of medical schools in Australia outlines the social mission as seen by the Deans of medical schools and concentrates on two aspects: the improvement of rural health, and that of Indigenous Peoples (Aborigines and Torres Strait Islanders). | The social missions of Australian medical schools have been identified as providing quality staff for health care, increasing the number of general practitioners and rural doctors, and increasing access to medical training for Indigenous Peoples. |
| 2 | Quest for Social Accountability: experiences of a new health sciences university in Nepal. (Chapagain et al., 2000) | Nepal | B.P. Koirala Institute of Health Sciences | 2000 | To measure the school’s achievements in responding to societal needs. | Useful in identifying the school’s strengths as well as weaknesses in responding to societal needs. |

| 3 | Perceptions of faculty toward “Social obligation” at an Indian medical school. (Dandekar et al., 2021) | India | Seth G. S. M. C. and K. E. M. Hospital, Mumbai. | 2021 | To explore the knowledge and perception of faculty members towards social obligation. | This study illustrates how the faculty of an Indian medical school perceive the subject of social obligation. Although faculty share similar perceptions on many aspects of social, and cultural values and partnerships, they have different ideas on what social obligation means, how it is likely to be implemented, how it may succeed, and what challenges may be faced. |
| --- | --- | --- | --- | --- | --- | --- |
| 4 | Translating medical school social missions to student experiences. (Ellaway et al., 2018) | Australia | Flinders University, James Cook University | 2018 | To explore how the social mission of different medical schools translated to the experiences of its graduating medical students. | Whether a medical school’s social mission is focused on equity of access to the medical profession or its graduates serving community needs, the mission principles need to be translated into practice. This translation process involves a series of externalization and internalization steps, each of which determines how much and what aspects of the mission are translated. |
| 5 | Practice intentions at entry to and exit from medical schools aspiring to SA: findings from the Training for Health Equity Network Graduate Outcome Study. (Larkins et al., 2018) | Australia | Flinders University, James Cook University | 2018 | To describe characteristics associated with intention to practice with underserved populations for entry and exit cohorts at THEnet medical schools, and to consider student characteristics associated with differences in practice intention across different contexts. | These data confirm that rurality of origin and other measures of disadvantage predict intention to work with underserved populations across eight medical schools on five continents. The intention to practice with underserved communities is similar or higher upon exit from the program. These cross-sectional findings suggest that the student respondents’ experience throughout the program is training and socializing graduates towards meeting population needs. |
| 6 | Measuring SA in health professional education: Development and international pilot testing of an evaluation framework. (Larkins et al., 2013) | Australia | James Cook University, Flinders University | 2013 | To describe the development of THEnet evaluation framework for socially accountable health professional education presents the framework to be used as a tool by other schools and discusses the findings of pilot implementation at five schools. | THEnet evaluation framework is applicable and useful across contexts. It is possible and desirable to assess progress towards SA in health professional schools and this is an important step in producing health professionals with knowledge, attitudes, and skills to meet the challenges of priority health needs of underserved populations. |
| 7 | Complexity or meaning in health professional education and practice? (Lowe, 2014) | Australia | University of Southampton | 2014 | To explore the meaning of complexity by asking how health professionals are educated and some of the consequences of that education. | Health professional education discourses could be seen as obscuring broader issues that impact healthcare practice. Health professional curricula occupy a privileged location within society and as such need to provide a socially accountable practice. Ways to develop socially accountable practices are explored within the context of the widening gap between disadvantaged and advantaged people on a global level. |
| 8 | How SA Can be Incorporated into an Urban Community‑based Medical Education Program: An Australian Initiative Education for Health. (Mahoney et al., 2014) | Australia | Flinders University | 2014 | To describe an initiative that translates this broad intent into meaningful activities that foster positive attitudes to SA among medical students in the context of an already full curriculum. | A model for incorporating SA into the medical curriculum with sustainable activities that benefit the community and medical students was described. Further research and evaluation of the impact on both the community group involved and on medical students is essential. |
| 9 | Building blocks for SA: a conceptual framework to guide medical schools. (Preston et al., 2016a) | Australia | James Cook University | 2016 | To present a conceptual framework, derived from empirical research from practice, describing the building blocks for socially accountable medical education. | Many of the building blocks are like those conceptualized in SA theory hence, this conceptual framework is informed by what happens in practice-empirical evidence rather than prescriptions. Consequently, it is valuable in that it puts some theoretical thinking around everyday practice in specific contexts, addressing a gap in the medical education literature. The building blocks framework includes guidelines for socially accountable practice that can be applied at policy, school, and individual levels. |
| 10 | From personal to global: Understandings of SA from stakeholders at four medical schools. (Preston et al., 2016b) | Australia | James Cook University | 2016 | To address the question of how SA is conceptualized by staff, students and community members associated with four medical schools aspiring to be socially accountable in two countries. | The assumption that SA is universally understood could not be confirmed from these data. To strengthen SA, it is useful to learn from these institutions’ experiences to contribute to the development of the theory and practice of activities within socially accountable medical schools. |
| 11 | Best Practices in Community-Oriented Health Professions Education: International Exemplars Education for Health. (Richards, 2001) | Multi sites (India) | University of Illinois at Chicago | 2001 | To identify key issues in designing and implementing community-based education | Despite their differences, all nine exemplars are engaged in processes of organizational change both in terms of how one teaches and where that teaching occurs. Significant change calls for nothing less than rewarding faculty members for doing things differently and thereby developing different expectations of their students. Schools are becoming more community-oriented and socially accountable, and all these programs have accepted two fundamental tenets: ``take public money, give to the public’’ and ``place matters’’. |
| 12 | The Training for Health Equity Network Evaluation Framework: A Pilot Study at Five Health Professional Schools.(Ross et al., 2014) | Multi sites  (Australia) | James Cook University |  | To describe the pilot implementation of testing the evaluation framework across five THEnet schools and examine whether the evaluation framework was practical and feasible across contexts for critical reflection and continuous improvement in terms of progress towards SA. | This pilot study has demonstrated that the evaluation framework is a practical and useful tool and a whole of school reflective process for health professional schools to assess their progress toward SA. The study has informed THEnet’s research activities, which will ultimately contribute to strengthening the evaluation framework. |
| 13 | Measuring social responsiveness of medical schools: a case study from Thailand. (N, 1999) | Thailand | Chulalongkorn University | 1999 | To conduct a program evaluation on the social responsiveness of a medical school in Thailand. | The social responsiveness, using the SA grid, was found to be outstanding in the educational domain, good in-service domain, and fair in the research domain. The leadership, participation and loyalty of the staff, the missions of the medical schools and the hospital were identified as the keys to success. |
| 14 | Does a socially accountable curriculum transform health professional students into competent, work-ready graduates? A cross-sectional study of three medical schools across three countries. (Woolley et al., 2019) | Multi sites (Australia) | James Cook University | 2019 | To assess the perspective of clinical supervisors regarding the clinical and ‘socially accountable’ competencies, work-readiness, and fit-for-purpose to practice in the local region, of first-year medical graduates from three socially accountable medical schools across three countries. | The study suggests that SAHPE produces a local medical work force that is at least as ‘work ready’ as graduates from more traditional medical schools in terms of overall performance and biomedical knowledge and skills but rated much higher in socially accountable competencies such as communication skills, working as a team, professionalism, commitment to health equity, and working with under-served populations. In addition, identifying the knowledge and competency areas for improvement in SAHPE graduates can inform the curriculum of socially accountable schools for continued development. |
| 15 | Work settings of the first seven cohorts of James Cook University Bachelor of Medicine, Bachelor of Surgery graduates: Meeting an SA mandate through contribution to the public sector and Indigenous health services. (Woolley et al., 2018) | Australia | James Cook University | 2018 | The James Cook University medical school’s mission is to produce a workforce appropriate for the health needs of northern Australia. | James Cook University medical graduates appear to work in a higher proportion of public settings; in particular, primary care settings, than Australian medical graduates. This is an appropriate mix for the predominantly rural and remote geography of Queensland and its associated medical workforce priorities. |
