# Supplementary material for: Making Medical Education Socially Accountable in Australia and Southeast Asia: A Systematic Review
Source: Med Sci Educ. 2025 Feb 25;35(3):1767–76. doi: 10.1007/s40670-025-02322-x (PMC12228936; doi:10.1007/s40670-025-02322-x)
Supplement: Supplementary file 2 — Supplementary file2 (DOCX 18 KB) [file 40670_2025_2322_MOESM2_ESM.docx]

**Supplementary Document 2: Quality assessment for included studies**

|  | Study 1 | Study 2 | Study 3 | Study 4 | Study 5 | Study 6 | Study 7 | Study 8 | Study 9 | Study 10 | Study 11 | Study 12 | Study 13 | Study 14 | Study 15 |
| --- | --- | --- | --- | --- | --- | --- | --- | --- | --- | --- | --- | --- | --- | --- | --- |
| Are the questions/hypotheses stated clearly and relevant to the research questions? | Y | Y | Y | Y | Y | Y | Y | Y | Y | Y | Y | Y | Y | Y | Y |
| What is the study design and is it appropriate for the research question? | Qual; yes | Quant; yes | Qual; yes | Qual; yes | Quant; yes | Qual; yes | Qual; yes | Qual; yes | Qual; yes | Qual; yes | Qual; yes | Mixed; yes | Qual; yes | Quant; yes | Quant; yes |
| How were participants selected and is the population appropriate? | Purposive; yes | Purposive; yes | Purposive; yes | Purposive; yes | Purposive; yes | Purposive; yes | Purposive; yes | Purposive; yes | Purposive; yes | Purposive; yes | Purposive; yes | Purposive; yes | Purposive; yes | Purposive; yes | Purposive; yes |
| Has the impact on the sample population been presented? | Y | Y | Y | Y | Y | Y | Y | Y | Y | Y | Y | Y | Y | Y | y |
| Did the author answer the study question appropriately? | Y | Y | Y | Y | Y | Y | Y | Y | Y | Y | Y | Y | Y | Y | Y |
| Are the studies' strengths highlighted? | Y | N | Y | Y | Y | Y | Y | Y | Y | Y | Y | Y | Y | Y | Y |
| Are the limitations discussed with suggested steps that may improve future results? | Y | Y | Y | Y | Y | Y | Y | Y | Y | N | Y | N | N | Y | Y |
| Did they suggest further study they will undertake? | N | Y | N | Y | Y | Y | Y | Y | Y | Y | N | Y | N | N | N |

Qual: Qualitative, Quan: Quantitative
